# Biomass Allocation Patterns Are Linked to Genotypic Differences in Whole-Plant Transpiration Efficiency in Sunflower

**DOI:** 10.3389/fpls.2017.01976

**Published:** 2017-11-17

**Authors:** Luciano Velázquez, Ignacio Alberdi, Cosme Paz, Luis Aguirrezábal, Gustavo Pereyra Irujo

**Affiliations:** ^1^Laboratorio de Fisiología Vegetal, Unidad Integrada Balcarce, Instituto Nacional de Tecnología Agropecuaria - Universidad Nacional de Mar del Plata, Balcarce, Argentina; ^2^Consejo Nacional de Investigaciones Científicas y Técnicas, Buenos Aires, Argentina

**Keywords:** *Helianthus annuus*, sunflower, biomass allocation, transpiration efficiency, genotypic variability

## Abstract

Increased transpiration efficiency (the ratio of biomass to water transpired, TE) could lead to increased drought tolerance under some water deficit scenarios. Intrinsic (i.e., leaf-level) TE is usually considered as the primary source of variation in whole-plant TE, but empirical data usually contradict this assumption. Sunflower has a significant variability in TE, but a better knowledge of the effect of leaf and plant-level traits could be helpful to obtain more efficient genotypes for water use. The objective of this study was, therefore, to assess if genotypic variation in whole-plant TE is better related to leaf- or plant-level traits. Three experiments were conducted, aimed at verifying the existence of variability in whole-plant TE and whole-plant and leaf-level traits, and to assess their correlation. Sunflower public inbred lines and a segregating population of recombinant inbred lines were grown under controlled conditions and subjected to well-watered and water-deficit treatments. Significant genotypic variation was found for TE and related traits. These differences in whole-plant transpiration efficiency, both between genotypes and between plants within each genotype, showed no association to leaf-level traits, but were significantly and negatively correlated to biomass allocation to leaves and to the ratio of leaf area to total biomass. These associations are likely of a physiological origin, and not only a consequence of genetic linkage in the studied population. These results suggest that genotypic variation for biomass allocation could be potentially exploited as a source for increased transpiration efficiency in sunflower breeding programmes. It is also suggested that phenotyping for TE in this species should not be restricted to leaf-level measurements, but also include measurements of plant-level traits, especially those related to biomass allocation between photosynthetic and non-photosynthetic organs.

## Introduction

Drought is one of the major limitations to crop yields worldwide, and global climate change scenarios predict a future increase in the risk of drought (Dai, 2012). Moreover, a growing world population increases the demand of water for human consumption. In this context, a lower priority for agricultural water could be expected (Tardieu, 2005).

Increased transpiration efficiency (the ratio of biomass to water transpired) could then represent an interesting trait to achieve a more conservative use of water (Tardieu and Tuberosa, 2010) and improving drought tolerance under some water deficit scenarios (Tardieu, 2011). Targeting only increased transpiration efficiency could, however, lead to negative results in breeding programs. It has often resulted in plants with lower biomass and yield because of reduced transpiration but also lower photosynthesis (Blum, 2005). Dissecting the mechanisms underlying transpiration efficiency in a given species has been proposed as a novel approach to attain increased transpiration efficiency without compromising biomass and photosynthesis, which is crucial for breeding programs (Tardieu and Tuberosa, 2010).

The term transpiration efficiency (TE) is used to define different expressions such as the ratio of photosynthesis to stomatal conductance (intrinsic TE) and the ratio of biomass to transpiration in a plant (whole-plant TE). Intrinsic TE is usually considered as the primary source of variation in whole-plant TE. However, intrinsic TE is not always a reliable estimator of whole-plant TE (e.g., Tomás et al., 2012).

Transpiration efficiency (either intrinsic or whole-plant) depends on physiological traits of the plant and on the environment. Sinclair (2012) mentions environmental variables such as CO_2_ concentration and atmospheric vapor pressure deficit (VPD), and physiological traits such as leaf photosynthetic capacity, biochemical composition of the plant biomass, and hydraulic limitations to water flow in the plant. The physiological traits considered by Sinclair (2012) are consistent with those most frequently investigated in relation to intrinsic TE, which are those related to the fluxes of water and carbon between the leaf and the atmosphere. Carbon isotope discrimination (CID), a reliable estimator of intrinsic TE used frequently for phenotyping (Tardieu, 2011; Masuka et al., 2012), has frequently been shown to be only poorly correlated to whole-plant TE in different species (e.g., Krishnamurthy et al., 2007; Devi et al., 2011; Adiredjo et al., 2014a). If the response of water fluxes to environmental variables are, however, studied at whole-plant level, a better correlation to whole-plant TE is usually found (e.g., Devi et al., 2011). Moreover, in sunflower, genetic evidence clearly show that, while there are genetic regions associated both to CID and whole-plant TE, there are genetic regions associated independently to each of both variables (Adiredjo et al., 2014b).

The integration of organ-level traits into whole-plant phenotypes is key to plant adaptation (Mason et al., 2017), and thus biomass allocation could play an important role in determining whole-plant TE, as it can be inferred from results of some works (e.g., Liu and Stützel, 2004). Changes in carbon allocation between different plant organs are usual responses to different environmental variables, including water availability, across plant species (Poorter and Nagel, 2000). There is, however, very limited information regarding how genotypic variation in carbon allocation could impact TE in a given species and in sunflower in particular. In a theoretical framework in which carbon lost through night-time respiration or in non-photosynthetic organs (e.g. roots) and water lost independently of carbon uptake (e.g., non-stomatal transpiration) could contribute to explain the differences between intrinsic and whole-plant TE (Rasheed et al., 2013), it would be expected that increased allocation of biomass to non-photosynthetic organs would decrease whole-plant TE. Nevertheless, TE has been shown to be positively associated to root biomass in some species (e.g., Van den Boogaard et al., 1997; Puangbut et al., 2009). Stem photosynthesis, on the other hand, is significant in many asteraceae (Rawson and Constable, 1980; Nilsen, 1999), suggesting that sunflower stems could be a useful source of carbon, especially in early stages of development under high irradiance.

Sunflower is considered as moderately tolerant to water stress, presenting several traits related to drought tolerance such as a deep root system, temporary wilting, and a stable harvest index as compared to other crops (Andrade and Sadras, 2000). On the other hand, it shows other characteristics which could be considered unfavorable under drought, such as a transpiration rate which is higher (Hattendorf et al., 1988) and less responsive to soil water deficits (Connor and Sadras, 1992) or evaporative demand (Camacho-B et al., 1974) than in other crop species. Breeding for a more conservative water use in this species, especially when water is limiting, could therefore be a desirable target. Achieving this goal without reducing yield would require also increasing transpiration efficiency. Sunflower has a significant variability in TE (Lambrides et al., 2004) and CID (Adiredjo et al., 2014b), which could allow for this strategy. Although it is said that the TE has been considered as a constitutive trait (Kholová et al., 2010 in pearl millet; Slafer et al., 2005; Slafer and Araus, 2007 in wheat; Pereyra-Irujo et al., 2012 in soybean), a significant genotype × water scenario interaction has been recently reported for TE and CID in sunflower (Adiredjo et al., 2014b).

A better knowledge of the effect of leaf and plant-level traits on TE could be helpful to obtain a more comprehensive knowledge of the determination of this trait in sunflower, and to aid in detecting more efficient genotypes for water use. The objective of this study was, therefore, to assess if genotypic variation in whole-plant TE under well-watered and water deficit conditions is better related to leaf-level traits (e.g., carbon isotope discrimination, transpiration rate per unit leaf area) or plant-level traits (e.g., biomass allocation to shoots, leaves, and roots).

## Materials and methods

Three experiments were conducted using sunflower public inbred lines and a population of recombinant inbred lines (RIL). The inbred lines used included HAR2 and HA64, which had shown a contrasting response of leaf growth to water deficit (Pereyra-Irujo et al., 2008) and HA89 and RHA801, which had previously shown contrasting values of TE (Lambrides et al., 2004). The RIL population is comprised of F_8_ seeds resulting from six cycles of selfing by single-seed descent from a segregating population of F_2_ plants, derived from the cross of HAR2 and HA64.

Experiment 1 was aimed at verifying the existence of variability in whole-plant TE and possibly related whole-plant and leaf-level traits in the initial group of four inbred lines, in plants subjected to well-watered (WW) and water deficit (WD) conditions. Experiment 2 included 71 RILs from the HAR2 × HA64 population, and was aimed mainly at assessing the genetic variability in TE under WW and WD conditions. In experiment 3, 23 randomly selected RILs from the same population were grown under WD conditions, and traits underlying whole-plant TE were analyzed.

In all experiments, the intensity of the soil water deficit reached was mild (−0.65 MPa), and daily adjustment of soil water content ensured that all genotypes were subjected to a similar water deficit treatment irrespective of their leaf area or water use rate, which has been shown to increase the repeatability of rankings of genotype performance under drought (Pereyra-Irujo et al., 2007).

### Plant culture

In experiment 1, 16–20 plants of each of the inbred lines HA64, HAR2, HA89, and RHA801 were sown in 2.75 L pots filled with 2,400 g of soil (typic Argiudoll, horizon A), and arranged following a completely randomized design. Soil water content was measured initially by oven-drying soil samples at 105°C for 48 h, and then monitored daily by weighing and watering of the pots. Plants were grown without water limitations (−0.05 MPa) for 9 days in a growth chamber (16 h photoperiod, 500 μmol.m^−2^.s^−1^ PAR, 23°C, 40% RH, 1.4 kPa vapor pressure deficit, VPD). On day 10 after sowing, plants were transferred to an automatic phenotyping platform (GlyPh, Pereyra-Irujo et al., 2012) located in a greenhouse (11 h photoperiod, 570 μmol.m^−2^.s^−1^ PAR, 22.5/16.0°C day/night, 1.3/0.8 kPa VPD day/night). Pots were weighed and watered automatically to a soil water content of 0.28 g water·g soil^−1^ (−0.05 MPa) daily until plants had 8–10 fully expanded leaves in all genotypes. At this moment (day 25 after sowing), two treatments were imposed: soil water content of half of the plants of each genotype was gradually decreased from 0.29 to 0.19 g water·g soil^−1^ (−0.65 MPa) during a period of 12 days (water-deficit treatment, WD), while the rest of the plants were watered to a constant soil water content of 0.29 g water·g soil^−1^ (−0.05 MPa, well-watered treatment, WW).

In experiment 2, 71 RILs and the parental lines HA64 and HAR2 were sown as in Experiment 1. Four plants of each RIL and 8 plants of the parental genotypes were grown in a greenhouse (12.2 h photoperiod, 480 μmol.m^−2^.s^−1^ PAR, 20°C, 0.9 kPa VPD; mean values), arranged following a complete block design. Pots were weighed and watered manually to a soil water content of 0.25 g water·g soil^−1^ (−0.05 MPa) daily until plants had 10 fully expanded leaves on average. At this moment (day 25 after sowing) two treatments were imposed: the soil water content of half of the plants of each genotype was decreased to 0.17 g water·g soil^−1^ (−0.65 MPa), maintained at that value during a period of 8 days, and finally increased to 0.25 g water·g soil^−1^ (−0.05 MPa, water-deficit treatment, WD) during 3 days; the rest of the plants were watered to a constant soil water content of 0.25 g water·g soil^−1^ (−0.05 MPa, well-watered treatment, WW).

In experiment 3, 23 randomly-selected RILs from the same population were sown as in Experiment 1. As in Experiment 1, four plants of each RIL were initially grown in a growth chamber (same conditions) and then transferred to the automatic phenotyping platform GlyPh in greenhouse (10.5 h photoperiod, 500 μmol.m^−2^.s^−1^ PAR, 17.5/16°C, 1.0/0.8 kPa VPD day/night), arranged following a complete block design. Pots were weighed and watered automatically to a soil water content of 0.29 g water·g soil^−1^ (−0.05 MPa) daily until plants had 10 fully expanded leaves on average. At this moment (day 19 after emergence), the soil water content of all the plants was gradually decreased from 0.29 to 0.19 g water·g soil^−1^ (−0.65 MPa) during a period of 14 days (water-deficit treatment, WD).

### Measurements

#### Environmental measurements

Air temperature, relative humidity and incident radiation were measured and recorded every 5 min using data loggers (Em50, Decagon devices, USA, in Experiment 1; Meteo, Cavadevices, Argentina, in Experiments 2 and 3).

#### Plant-level measurements

Total water use was measured daily for each pot, and plant water use was calculated by subtracting direct soil evaporation (measured in at least four pots with no plants in all experiments). At the end of each experiment, shoots (all three experiments) and roots (experiments 1 and 3) were harvested. Shoot were further separated into stems (plus petioles) and leaf blades. Dry mass of the samples was determined after oven-drying the samples at 60°C for 48 h. In experiments 1 and 3, final leaf area was measured by image analysis of photographs of sampled leaves (Image J, National Institute of Health, US). In experiment 2, leaf area was estimated through non-destructive leaf length and width measurements prior to harvest, as in Pereyra-Irujo et al. (2008).

Transpiration efficiency was calculated for each plant as the ratio between final dry mass and plant water use (total water use—soil evaporation). Shoot transpiration efficiency (TE_s_) was calculated for all three experiments using shoot dry mass; whole-plant transpiration efficiency (TE_wp_) was calculated using total plant dry mass (shoot + root) for experiments 1 and 3.

Carbon allocation between different organs was analyzed according to the framework used by (Poorter et al., 2012). Leaf area ratio (LAR, the amount of leaf area per unit shoot weight), specific leaf area (SLA, the amount of leaf area per unit leaf mass), and leaf mass fraction (LMF, the fraction of the plant biomass allocated to leaves) were calculated. The stem mass fraction (SMF) and root mass fraction (RMF, when roots were harvested) were also calculated. These variables are inter-related, so that LAR = SLA × LMF, and LMF + SMF + RMF = 1. Petioles were included in the stem fraction, as suggested by Poorter and Nagel (2000). LAR, LMF, and SMF were calculated for all experiments using shoot mass (LAR_s_, LMF_s_, and SMF_s_) or using whole-plant dry mass (shoot + root, LAR_wp_, LMF_wp_, and SMF_wp_) in experiments 1 and 3.

#### Leaf-level measurements

In experiments 1 and 3, transpiration rate per unit leaf area was determined at different moments during the drying period (5, 8, and 10 days after the start of the treatment in experiment 1; 12 and 13 days after the start of the treatment in experiment 3), by dividing the daily plant water used by the plant leaf area. At the end of the experiment, imprints from the middle portion of the lamina midway between the midrib and leaflet edge on the adaxial and abaxial side of the fourth leaf were taken by the acrilic varnish method (Fanourakis et al., 2014). Images of the imprints were acquired using an optical microscope (Olympus BX51TF, Tokyo, Japan) connected to a digital camera (Olympus Q17024, Qcolor 3, Olympus America, Canada). Stomatal density (i.e., number per unit leaf area) was counted on two non-overlapping interveinal fields of view per leaf at 200 × magnification. Image processing was performed with the ImageJ program (Schneider et al., 2012).

In experiment 1, leaf-level measurements were performed at the same three moments, on the fourth leaf of each plant, at noon. Stomatal conductance was measured using a porometer (SC-1, Decagon Devices, USA); leaf temperature was measured with an infrared thermometer (Omegaette os-fs, Omega, Canada); photochemical efficiency of Photosystem II was measured using a fluorometer (Fluorpen FP100, Photon Systems Instruments, Czech Republic).

In experiment 3, a quantitative measurement of leaf wilting was carried out based on the digital analysis of top-view images captured by the GlyPh platform. A wilting index was calculated as the ratio of the projected area of the plant at noon and the same area early in the morning, in a day with high VPD (1.5 Kpa, 3 days after the start of the treatment). In the same experiment, leaf samples of three plants of six selected genotypes which had shown differences in TE were used to determine carbon isotope discrimination (CID) as the ^13^C/^12^C ratio (δ^13^C), determined by EA-IRMS (Elemental Analyzer Isotope Ratio Mass Spectrometry) according to Coplen et al. (2006) at the Institute of Geochronology and Isotope Geology (INGEIS-CONICET, Buenos Aires, Argentina).

### Data analysis

For experiment 1 data, analyses of variance (ANOVA) were carried out with the R statistical package software (R Core Team, 2013). Data from experiments 2 and 3 was first analyzed jointly using the Proc MIXED of the SAS statistical software (SAS University Edition, 2017). The significance of the variance components (genotype, environment, and genotype × environment interaction) was tested using a likelihood ratio test comparing the full model with a reduced model lacking the factor to be tested (Holland et al., 2003). The broad sense heritability, which estimates the proportion of the total variance of a trait that is determined by the genotype, was calculated on a mean basis (along with its standard error) as in Holland et al. (2003), with the following equation:

$$h^{2}=\frac{\sigma_{g}^{2}}{\frac{\sigma_{e}^{2}}{rt}+\frac{\sigma_{ge}^{2}}{t}+\sigma_{g}^{2}}$$

Where $\sigma_{g}^{2}$ is the genotypic variance, $\sigma_{ge}^{2}$ is the genotype × environment variance, $\sigma_{e}^{2}$ is the experimental error, and t and r are the number of environments and replicates, respectively (Fehr, 1987).

Correlation analyses were then carried out to evaluate the association between TE and traits measured at leaf- and whole-plant-level, in the experiments involving the RIL population (experiments 2 and 3). Correlations were analyzed either using data from each experiment, or with the joint dataset. Phenotypic, genotypic, and environmental correlations (and their standard error) were calculated using multivariate restricted maximum likelihood estimation using the Proc MIXED of the SAS statistical software. The method implemented by Holland (2006) for calculating phenotypic and genotypic correlations was extended for also calculating the environmental correlation from the variances and covariances estimated with Proc MIXED.

## Results

### Genotypic variability for TE and related traits in four inbred lines

**Figure 1:**
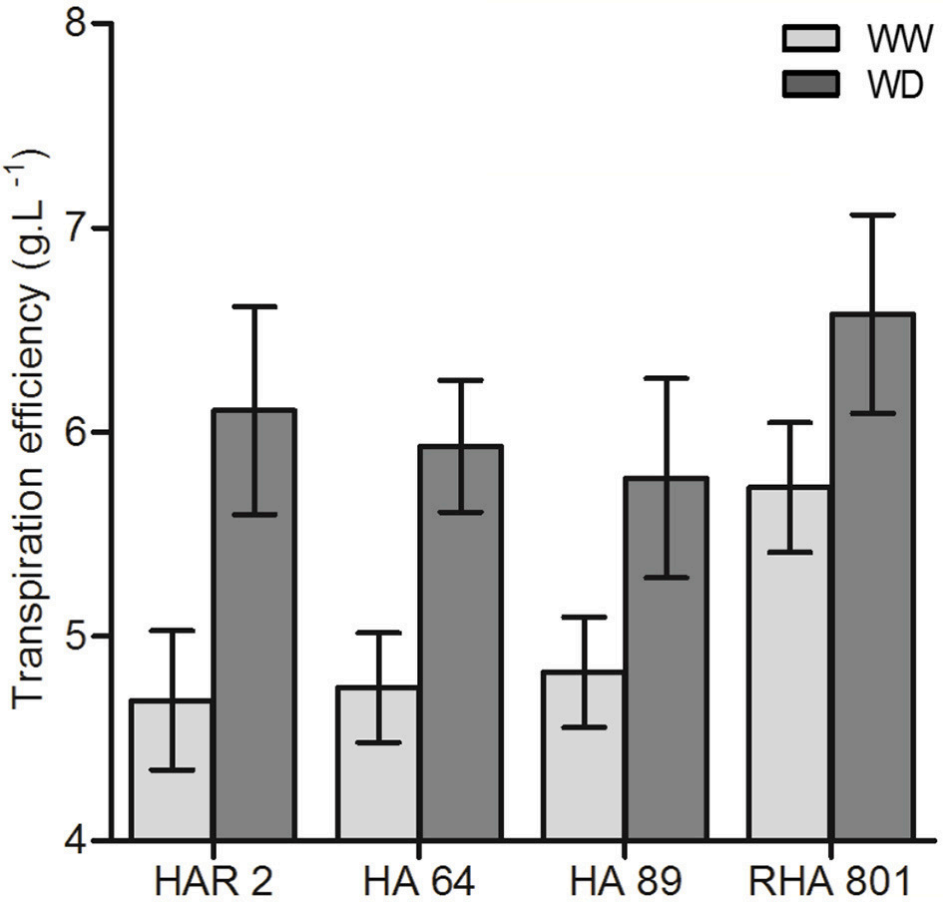
Whole plant (shoot + root) transpiration efficiency for the four evaluated lines under well-watered (WW) and water deficit (WD) conditions. Error bars represent the 95% confidence interval of the mean.

In experiment 1, where 4 inbred lines were studied, genotypic differences in whole-plant TE under well-watered conditions were found, ranging from 4.7 to 5.7 g L^−1^ (Figure 1). Under water deficit, TE increased up to values of 5.8 to 6.6 g L^−1^, equivalent to an increase of 15 to 30% for different genotypes (Figure 1), although the genotype × treatment interaction was not significant.

**Figure 2:**
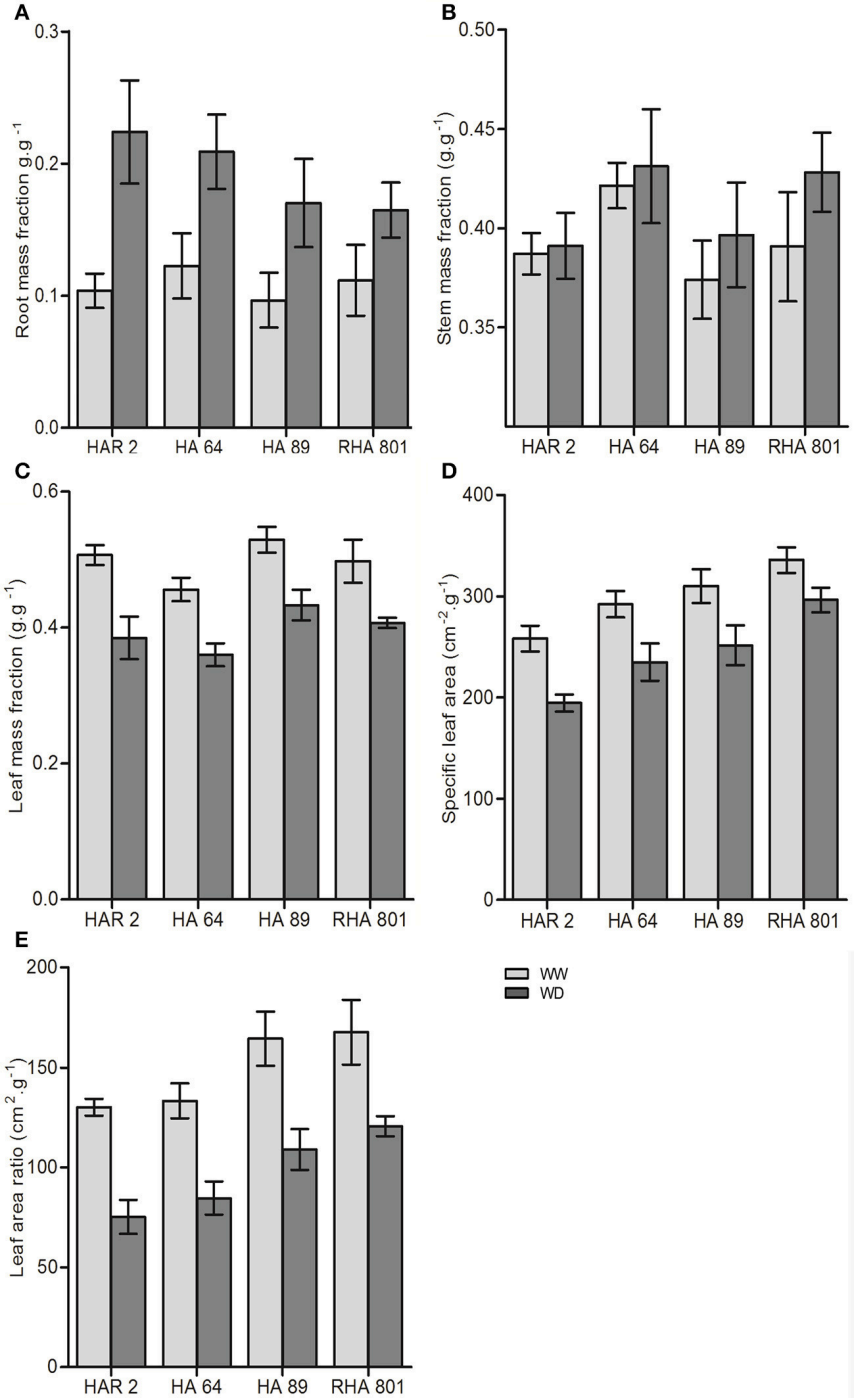
Whole-plant traits for the four evaluated lines under well-watered (WW) and water deficit (WD) conditions in Experiment 1: **(A)** root mass fraction, **(B)** stem mass fraction, **(C)** leaf mass fraction, **(D)** specific leaf area, and **(E)** leaf area ratio. Error bars represent the 95% confidence interval of the mean.

**Figure 3:**
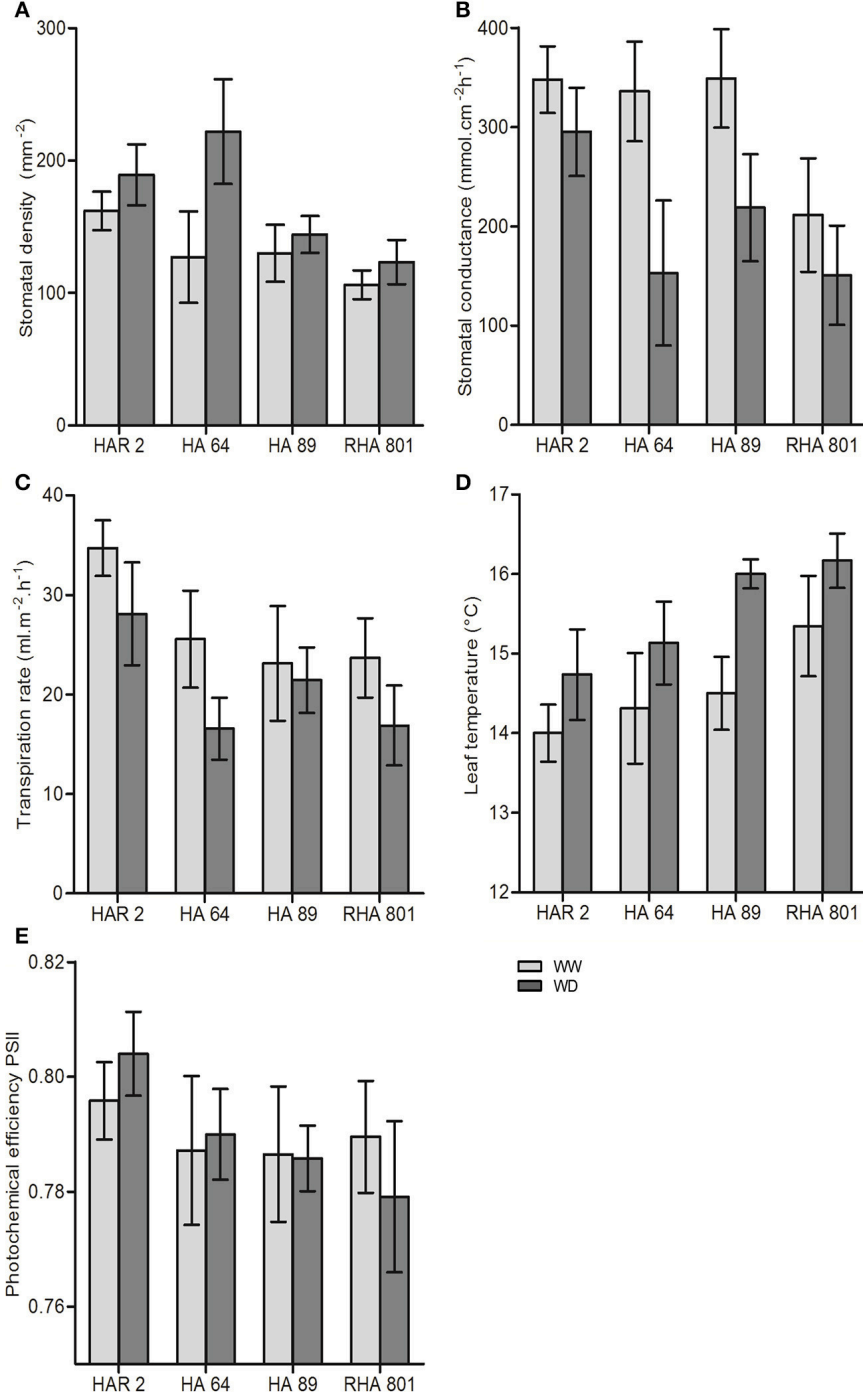
Leaf-level traits for the four evaluated lines under well-watered (WW) and water deficit (WD) conditions in Experiment 1: **(A)** stomatal density, **(B)** stomatal conductance, **(C)** transpiration rate, **(D)** leaf temperature, and **(E)** photochemical efficiency of photosystem II. Error bars represent the 95% confidence interval of the mean.

All the traits related to biomass allocation presented in Figure 2 showed significant variability among genotypes and a significant effect of water deficit. Water deficit significantly increased the root weight fraction (Figure 2A) and the stem mass fraction (Figure 2B), and decreased the leaf mass fraction (Figure 2C), the specific leaf area (Figure 3D), and the leaf area ratio (Figure 2E). There were genotypic differences in the magnitude of the change in biomass allocation under water deficit only for the root mass fraction (i.e., significant genotype × treatment interaction), which ranged from a 48% increase in genotype RHA801 to a 116% increase in HAR2. None of the other traits showed a significant genotype × treatment interaction that could indicate a differential response to water deficit between genotypes.

All leaf-level traits presented in Figure 3 showed significant variability among genotypes. Water deficit showed opposing effects on stomatal density (significantly increased, Figure 3A) and stomatal conductance (significantly decreased, Figure 3B). Both traits showed a significant genotype × treatment interaction, indicating a differencial response to water deficit among genotypes. The largest effect of water deficit was observed in genotype HA64, which showed a 75% increase in stomatal density, and a 54% decrease in stomatal conductance. Transpiration rate also significantly decreased (Figure 3C), and leaf temperature increased under water deficit (Figure 3D), but with no significant genotype × treatment interaction. On the other hand, the photochemical efficiency of Photosystem II was not significantly affected by water deficit (Figure 3E).

### Genotypic variability and heritability of TE in a segregating population

**Figure 4:**
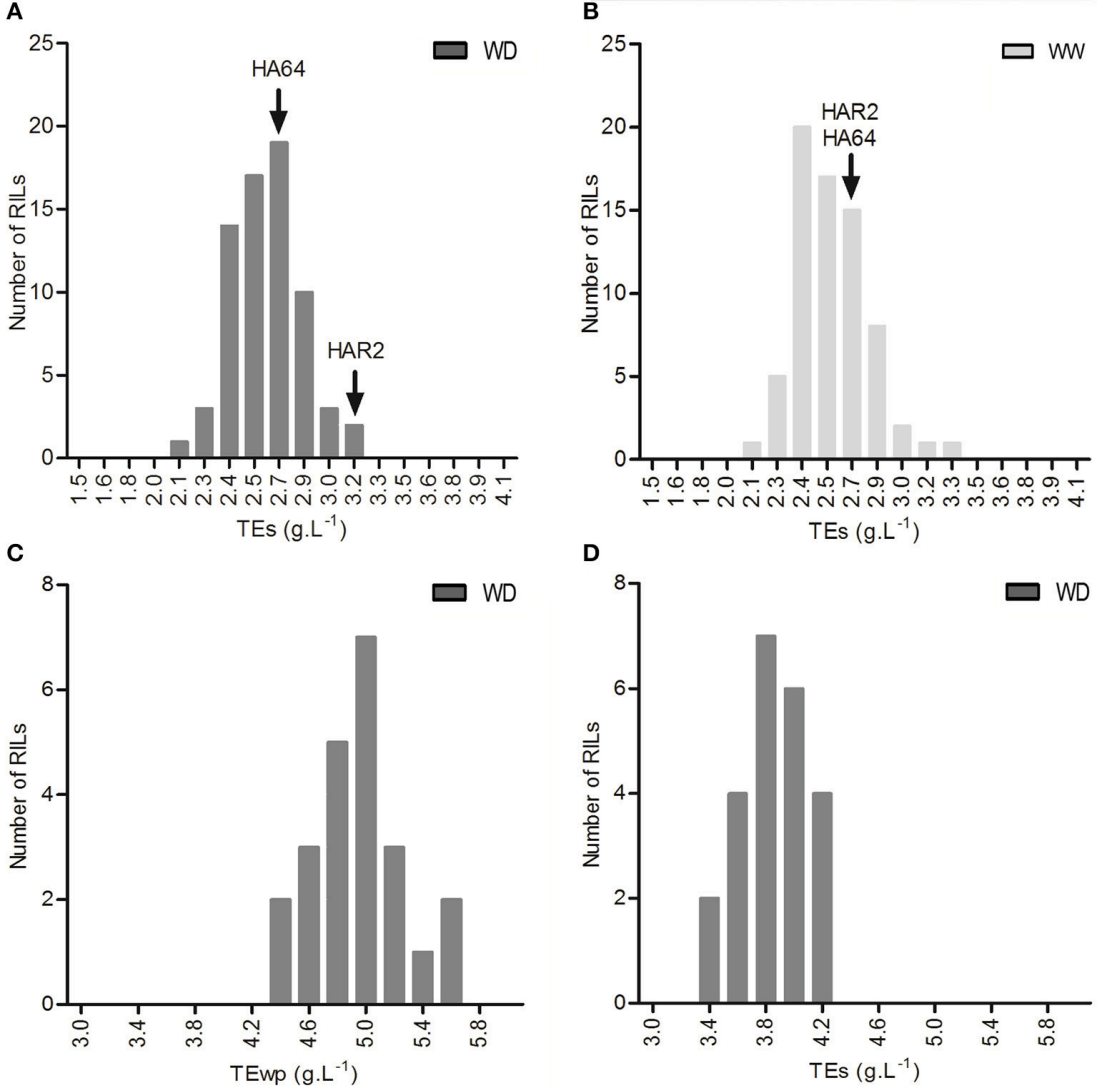
Frequency distribution of TE_s_ in experiment 2, **(A)** under well watered conditions, and **(B)** under water deficit. Frequency distribution of **(C)** TE_s_ and **(D)** TE_wp_, under water deficit conditions in experiment 3.

In experiment 2, a significant variability in TE was found both under well-watered and water deficit conditions among the 72 RILs, ranging from 1.9 to 3.3 g L^−1^ (well-watered) and 1.8 to 3.5 g L^−1^ (water deficit), but with no significant genotype × treatment interaction. This population showed transgressive segregation, as shown in Figures 4A,B. Experiment 3 also showed significant genotypic variation for TE (both for TE calculated using whole plant biomass, TE_wp_, and TE under drought calculated using shoot biomass, TE_s_) under drought among the 23 evaluated lines, with mean values ranging from 3 to 4.2 g L^−1^ for TEs (Figure 4C) and 4.2 to 6 g L^−1^ for TEwp (Figure 4D). A highly significant correlation was found TE_s_ and TE_wp_ in this experiment (Pearson's correlation coefficient between genotype means: *r* = 0.71, *p* < 0.001), and also in Experiment 1 (Pearson's correlation coefficient between genotype × treatment means: *r* = 0.90, *p* < 0.001).

Experiments 2 and 3 were analyzed jointly, considering the set of traits measured in both experiments and the 23 shared RILs. The analyses showed significant genotypic variance for all the evaluated traits, and a significant effect of the environment (Table 1). Despite the environmental or methodological variations between and within experiments, most traits showed a lack of significant genotype × experiment interaction, except for plant biomass and the specific leaf area. Broad-sense heritability showed intermediate to high values (0.52–0.86) for all traits, except for the specific leaf area (0.36, Table 1).

**Table 1:** Experiment means, range of best linear unbiased predicted means (BLUPs), significance of variance components, and broad sense heritability (and its standard error) for traits measured in the RIL population in experiments 2 (well-watered and water deficit treatments) and 3 (under water deficit).

**Trait (units)**	**Mean value**	**Range of predicted genotype means**	**Significance of variances**			***h^2^***
			**Genotypic**	**Environmental**	**G×E**	
LMF_s_ (g g^−1^)	0.60	0.52–0.67	<0.01^*^	<0.01^*^	0.40 ns	0.86 (0.04)
LAR_s_ (cm^2^ g^−1^)	143	116–175	<0.01^*^	<0.01^*^	0.09 ns	0.74 (0.09)
Water use (mL)	897	472–1303	<0.01^*^	<0.01^*^	1.00 ns	0.73 (0.07)
Leaf area (cm^2^)	386	225–534	<0.01^*^	<0.01^*^	0.08 ns	0.63 (0.13)
Shoot biomass (g)	2.77	1.55–4.17	<0.01^*^	<0.01^*^	<0.01^*^	0.60 (0.12)
TE_s_ (g L^−1^)	3.03	2.60–3.67	<0.01^*^	<0.01^*^	0.13 ns	0.52 (0.15)
SLA (cm^2^ g^−1^)	237	202–286	<0.01^*^	<0.01^*^	<0.01^*^	0.36 (0.22)

* *and ns indicate that variances are significantly different or not significantly different from zero, respectively*.

### Correlations between TE and related traits in a segregating population

The correlation between TE and other possibly related traits was analyzed in each of the experiments involving the RIL population, and also jointly using the set of shared traits and genotypes. First, the correlation between TE and its components (plant biomass and water use) was explored. Phenotypic correlation between TE and total plant water use was found to be not significant in all cases (Table 2). Nevertheless, a significant positive genotypic correlation was found only when both experiments were analyzed jointly, while a negative environmental correlation was observed only in experiment 2. The phenotypic correlation between TE and plant biomass was significant and positive in all cases (Table 2). Significant genotypic and environmental correlations were found when experiments were analyzed jointly. On the other hand, for leaf-level trait measured in experiment 3, no significant phenotypic, genotypic or environmental correlation was found between TE and carbon isotope discrimination, stomatal density, transpiration rate, or wilting index (Table 3).

**Table 2:** Phenotypic, genotypic, and environmental correlation coefficients (and their standard error) between TE and its components, water use and plant biomass (B).

**Traits**	**Experiment**	**Correlation coefficients**		
		**Phenotypic**	**Genotypic**	**Environmental**
TE_s_ vs. water use	2	−0.09 (0.07) ns	0.72 (0.44) ns	−0.29 (0.08)^*^
TE_s_ vs. water use	2–3	0.05 (0.07) ns	0.45 (0.22)^*^	−0.20 (0.11) ns
TE_wp_ vs. water use	3	−0.09 (0.15) ns	0.04 (0.31) ns	−0.21 (0.26) ns
TE_s_ vs. B_s_	2	0.35 (0.06)^*^	0.60 (0.24)^*^	0.12 (0.08) ns
TE_s_ vs. B_s_	2–3	0.45 (0.05)^*^	0.63 (0.18)^*^	0.28 (0.09)^*^
TE_wp_ vs. B_wp_	3	0.31 (0.14)^*^	0.34 (0.26) ns	0.28 (0.27) ns

*Subscripts indicate whether these variables were calculated using shoot biomass (s) or whole-plant biomass (wp). Data corresponds to Experiments 2 and 3, or to the joint analysis of both experiments*.

* *and ns indicate that correlation coefficients are significantly different or not significantly different from zero, respectively*.

**Table 3:** Phenotypic, genotypic, and environmental correlation coefficients (and their standard error) between whole-plant TE (TE_wp_) and leaf-level traits.

**Traits**	**Experiment**	**Correlation coefficients**		
		**Phenotypic**	**Genotypic**	**Environmental**
TE_wp_ vs.carbon isotope discrimination	3	−0.19 (0.21) ns	0.11 (0.60) ns	−0.34 (0.27) ns
TE_wp_ vs. stomatal density	3	−0.01 (0.14) ns	−0.20 (0.33) ns	0.12 (0.24) ns
TE_wp_ vs. transpiration rate	3	−0.17 (0.15) ns	−0.24 (0.27) ns	−0.10 (0.40) ns
TE_wp_ vs. wilting index	3	0.05 (0.15) ns	0.08 (0.30) ns	0.02 (0.29) ns

Data corresponds to experiment 3 under water deficit treatment. ^*^ and ns indicate that correlation coefficients are significantly different or not significantly different from zero, respectively.

Significant phenotypic correlations with TE were found for most biomass allocation traits (Table 4). The phenotypic correlation between TE and the leaf area ratio and the leaf mass fraction was found to be significant and negative in both experiments, and whether TE was calculated based on shoot or whole-plant data. On the other hand, the specific leaf area and the stem mass fraction were found to be significantly correlated to shoot-based TE, but neither of them nor the root mass fraction (measured only in Experiments 3) were phenotypically correlated to whole-plant based TE (Table 4). These correlations were then partitioned between the genotypic correlation and environmental correlation. Whenever correlations were found to be significantly different from zero for a trait, the sign of the correlation did not differ between phenotypic, genotypic and environmental correlation, or if it corresponded to whole-plant or shoot values (Table 4).

**Table 4:** Phenotypic, genotypic, and environmental correlation coefficients (and their standard error) between TE and either specific leaf area (SLA), leaf area ratio (LAR), leaf mass fraction (LMF), stem mass fraction (SMF), or root mass fraction (RMF).

**Traits**	**Experiment**	**Correlation coefficients**		
		**Phenotypic**	**Genotypic**	**Environmental**
TE_s_ vs. SLA	2	−0.35 (0.06)^*^	−0.67 (0.28)^*^	−0.35 (0.07)^*^
TE_s_ vs. SLA	2–3	−0.34 (0.06)^*^	−0.25 (0.39) ns	−0.39 (0.08)^*^
TE_wp_ vs. SLA	3	−0.14 (0.15) ns	0.18 (0.31) ns	−0.45 (0.31) ns
TE_s_ vs. LAR_s_	2	−0.44 (0.05)^*^	−0.73 (0.24)^*^	−0.40 (0.07)^*^
TE_s_ vs. LAR_s_	2–3	−0.49 (0.06)^*^	−0.65 (0.17)^*^	−0.46 (0.08)^*^
TE_wp_ vs. LAR_wp_	3	−0.51 (0.11)^*^	−0.42 (0.24) ns	−0.62 (0.22)^*^
TE_s_ vs. LMF_s_	2	−0.26 (0.06)^*^	−0.43 (0.23) ns	−0.24 (0.10)^*^
TE_s_ vs. LMF_s_	2–3	−0.36 (0.07)^*^	−0.55 (0.16)^*^	−0.25 (0.11)^*^
TE_wp_ vs. LMF_wp_	3	−0.51 (0.11)^*^	−0.80 (0.16)^*^	−0.24 (0.27) ns
TE_s_ vs. SMF_s_	2	0.26 (0.06)^*^	0.43 (0.23) ns	0.24 (0.10)^*^
TE_s_ vs. SMF_s_	2–3	0.36 (0.07)^*^	0.55 (0.16)^*^	0.25 (0.11)^*^
TE_wp_ vs. SMF_wp_	3	0.27 (0.14) ns	0.57 (0.23)^*^	−0.04 (0.35) ns
TE_wp_ vs. RMF_wp_	3	0.24 (0.14) ns	0.26 (0.30) ns	0.22 (0.22) ns

*Subscripts indicate whether these variables were calculated using shoot biomass (s) or whole-plant biomass (wp). Data corresponds to Experiments 2 and 3, or to the joint analysis of both experiments*.

* *and ns indicate that correlation coefficients are significantly different or not significantly different from zero, respectively*.

## Discussion

Significant genotypic variation was found for TE in sunflower genotypes, both in a group of four inbred lines, and in a population of RILs derived from the cross between two of them, as found previously for this species (Virgona et al., 1990; Lambrides et al., 2004). The ranking for TE found for the two previously studied genotypes (HA89 and RHA801) was similar to that found by Lambrides et al. (2004).

In several species, TE has been suggested to be a constitutive trait (i.e., genotype rankings are similar under different soil water contents; e.g., Virgona et al., 1990, in sunflower; Earl, 2002 and Pereyra-Irujo et al., 2012 in soybean; Rizza et al., 2012, in wheat). Therefore, the possibility of phenotyping to be performed under well-watered conditions has been suggested, which would simplify the experimental procedures for measurement and reduce treatment-induced error. In our study, no significant genotype × treatment interaction was found, either within or across experiments. These results contradict those of Adiredjo et al. (2014b), who found a significant genotype × water scenario interaction for TE, which would indicate that the evidence available so far for this species is not sufficient to conclude that this trait is constitutive. Moreover, Sherrard et al. (2009) found that genotypic correlations between traits can be significantly altered by water deficit. Based on this, it could be convenient that phenotyping for whole-plant TE and associated traits in sunflower be carried out under the drought scenario at which the breeding program is aimed.

Blum (2005) warns about most increases in TE being achieved through traits which reduce yield potential. In the RIL population analyzed in the present work, not only no evidence of a negative correlation of TE to shoot or total biomass was found, but the opposite trend was observed. However, TE was found to be associated to reduced biomass allocation to leaves and reduced leaf area per unit leaf biomass, which could be expected to lead to a reduction in biomass accumulation. Water deficit treatments imposed in our experiments were short in relation to the complete growth cycle of the plants. In the event of a stress of a longer duration, these traits could compromise carbon assimilation and yield potential.

TE was also found to be positively associated to the proportion of biomass allocated to stems. This kind of relationship has been found for other species, either by comparing different genotypes (e.g., Puangbut et al., 2009 in peanut) or different environments (e.g., Masle and Farquhar, 1988 in wheat). Even though increased biomass allocation to stems at the expense of leaf biomass could, as mentioned earlier, compromise carbon assimilation, it could also translate in increased reserves availability for remobilization during reproductive stages. Dosio et al. (2011) found that under water deficit, the concentration of soluble sugars increased in the developing capitulum, and proposed that upon rewatering these sugars are the basis for increased floret differentiation. Furthermore, Sadras et al. (1993) found that grain filling under drought conditions was strongly dependent on carbon flux from the stem. Hall et al. (1989) found that 27% of carbon allocated to grains in a droughted sunflower crop was originated in pre-flowering carbon stored in receptacle, stem and tap root (only 15% under well-watered conditions). These results suggest that increased biomass allocation to the stem under drought during vegetative stages could be beneficial for the availability of carbon for floret differentiation and grain filling at later stages.

Intrinsic TE is usually considered to be the main driver of whole-plant TE. There is the possibility of biomass allocation patterns associated with TE being only a consequence of intrisic TE-driven variation in whole-plant TE. However, correlation analyses in the segregating population showed that traits related to biomass allocation were more closely related to whole-plant TE than traits related to stomatal function (usually associated to intrinsic TE). Lambrides et al. (2004) and Adiredjo et al. (2014a) showed significant correlations between whole-plant TE and CID in sunflower, while a lack of relationship between CID and TE has been reported previously in other species (Hammer et al., 1997; Turner et al., 2007; Devi et al., 2011). In our study, the lack of correlation could be a consequence of the low variability present in the genotypes analyzed (1.0%0 range), as compared to those of previous studies: 4.4%0 (Lambrides et al., 2004), 3.4%0 (Adiredjo et al., 2014a), 2.5%0 (Virgona et al., 1990; Virgona and Farquhar, 1996). Unlike other studies in which the analyzed genotypes were selected for showing a wide range of CID, the low variability for this trait in our study may have allowed the detection of significant associations between TE and other traits.

A detailed analysis of whole-plant traits and TE showed variation in the magnitude of genotypic and environmental correlations. Genotypic correlations can arise from a linkage between genes underlying the studied traits or pleiotropic effects of the same gene or genes. These correlations cannot be generalized, since alleles and linkage desequilibrium between them can differ between populations of a species. On the other hand, environmental correlations can indicate the presence of physiological relationship between traits (Kibite and Evans, 1984; Cookson et al., 2005). In the present work, significant environmental correlations between traits of the same sign as the corresponding phenotypic and genotypic correlations were found for most traits. This indicates that even within groups of plants with the same genotype, variations in one trait are related to variations in the other trait, suggesting that these relationships could be of a physiological nature.

Significant negative correlations were found between TE and traits related to leaf area and leaf biomass allocation. Correlations between TE and SLA are consistant with the fact that SLA decreases as water becomes limiting in a wide range of species (Poorter et al., 2009). This trait showed, however, a high genotype × environment interaction and therefore a low heritability, which could be due to the fact that this trait is mostly a consequence of the independent variation in leaf expansion and biomass accumulation in leaves, which respond differently to environmental factors (Tardieu et al., 1999). Significant genotypic and environmental correlations were also found both between TE and LMF and between TE and LAR (which is expected since this variable is the product of SLA and LMF). In our experiments, LMF and LAR showed the highest heritability values and could be considered as promising traits for indirect selection for TE in sunflower, especially LMF due to its high genotypic correlation with whole-plant TE. A re-analysis of data presented by Lambrides et al. (2004) shows that in the group of 25 genotypes (inbred lines and hybrids) evaluated by them, whole-plant TE was significantly correlated to SLA (*r* = −0.58, *p* = 0.003^*^) and to LAR (*r* = −0.60, *p* = 0.002^*^); correlation with LMF was also negative but not significant (*r* = −0.38, *p* = 0.057ns). Despite some differences between the results obtained in both studies, which could be a consequence of different methods and environmental conditions (e.g., the work of Lambrides et al. (2004), was conducted under well-watered conditions only), these three interrelated traits (SLA, LAR, and LMF) seem to be consistently linked to TE in sunflower.

In this work only inbred (i.e., homozygous) genotypes were analyzed. This fact could represent a limitation, since most cultivated varieties of sunflower are hybrids and, therefore, studies using inbreds might not be able to capture the whole range of potential variability. Studies using inbred lines can, however, help shed light on the physiology of complex traits, and there are many examples in the literature in which this kind of studies have been carried out (e.g., Chimenti et al., 2002; Rauf and Saquat, 2008; among others). Lambrides et al. (2004) stated that studies using inbred lines can be useful for studying associations between traits but that these need to be confirmed in segregating populations, which was the approach followed in our study. Also, the experiments were carried out under controlled conditions with potted plants. This fact could also represent a limitation, since plants are grown under environmental conditions which largely differ from those found in the field. In spite of this, there are successful examples in the literature in which results found in greenhouse studies of drought tolerance were later confirmed in the field (e.g., Chapuis et al., 2012; Pardo et al., 2015). Future work should include testing the correlations observed in this study in hybrid genotypes grown in the field.

Surrogate traits most frequently used as proxies for TE are carbon isotope discrimination and canopy temperature, and also chlorophyll content and SLA as indirect estimates of photoynthetic capacity (Condon et al., 2004). All of these variables are measured at the leaf level, and only SLA could be a partial indicator of biomass allocation-driven variations in whole-plant TE. Based on our results, it is suggested that phenotyping for TE in sunflower should not be restricted to leaf-level measurements, but also include measurements of traits related to biomass allocation between photosynthetic and non-photosynthetic organs. The increased difficulty of these measurements should, however, be taken into account, since biomass allocation measurements require destructive sampling. This imposes a limitation to the use of these traits as selection criteria in breeding programmes, especially in early generations. This limitation could possibly be overcome through the use of proxies of biomass allocation traits (e.g., leaf area, plant height), and non-destructive image-based biomass measurements (e.g., Pereyra-Irujo et al., 2012).

## Conclusions

Differences in whole-plant transpiration efficiency between sunflower genotypes, and between plants within each genotype, were closely associated to biomass allocation patterns, and less closely associated to leaf-level traits. This association is probably of a physiological origin, and not only a consequence of genetic linkage in the studied population. Genotypic variation for biomass allocation could be potentially exploited as a source for increased transpiration efficiency in breeding programmes, independently of the frequently used intrinsic TE-related traits.

## Author contributions

GP and LA conceived and designed the experiments. LV and IA performed the experiments and measurements. CP performed leaf-level measurements. LV, IA, and GP analyzed the data. LV, IA, CP, LA, and GP discussed the results and wrote the manuscript.

### Conflict of interest statement

The authors declare that the research was conducted in the absence of any commercial or financial relationships that could be construed as a potential conflict of interest.

## Abbreviations

B: Biomass
B_s_: Biomass (shoot)
B_wp_: Biomass (whole-plant)
CID: Carbon isotope discrimination
LAR: Leaf area ratio
LAR_s_: Leaf area ratio (shoot)
LAR_wp_: Leaf area ratio (whole-plant)
LMF: Leaf mass fraction
LMF_s_: Leaf mass fraction (shoot)
LMF_wp_: Leaf mass fraction (whole-plant)
PAR: Photosynthetically active radiation
RIL: Recombinant inbred line
RMF: Root mass fraction
SLA: Specific leaf area
SMF: Stem mass fraction
SMF_s_: Stem mass fraction (shoot)
SMF_wp_: Stem mass fraction (whole-plant)
TE: Transpiration efficiency
TE_s_: Transpiration efficiency (shoot)
TE_wp_: Transpiration efficiency (whole-plant)
VPD: Vapor pressure deficit
WW: Well-watered
WD: Water deficit.
